# Concomitant Treatment with Etanercept and Tacrolimus Synergistically Attenuates Arthritis Progression via Inhibition of Matrix Metalloproteinase-3 Production and Osteoclastogenesis in Human TNF-*α* Transgenic Mice

**DOI:** 10.1155/2019/4176974

**Published:** 2019-12-17

**Authors:** Iwao Seki, Miwa Takai-Imamura, Tomomi Kohara-Tanaka, Satoshi Shirae, Minoru Sasano, Hiroaki Matsuno, Hiroyuki Aono

**Affiliations:** ^1^Research and Development Department, AYUMI Pharmaceutical Corporation, 8F Shijyo-Karasuma FT Square, 20 Naginatahoko-cho, Shimogyo-ku, Kyoto 600-8008, Japan; ^2^Matsuno Clinic for Rheumatic Disease, 7187-2, Kureha-cho, Toyama-shi, Toyama 930-0138, Japan

## Abstract

In the present study, we investigated the effects and mechanisms of action of a combined treatment with etanercept, a soluble tumor necrosis factor receptor (p75) Fc fusion protein, and tacrolimus, a calcineurin inhibitor on the progression of arthritis in human tumor necrosis factor-*α* (TNF-*α*) transgenic (hTNF-Tg) mice. Single-drug treatments with etanercept and tacrolimus attenuated the clinical signs but not the radiographic changes associated with the development of arthritis in mice. On the contrary, combined treatment significantly suppressed the radiographic progression and also improved the clinical signs. The combined treatment exhibited synergistic effects of the two drugs in reducing the serum matrix metalloproteinase-3 level and the number of peripheral CD11b^high^ osteoclast precursor cells. Moreover, tacrolimus inhibited the cytokine-induced osteoclast differentiation in synergy with etanercept in an *in vitro* assay. Interestingly, tacrolimus did not inhibit the production of antidrug antibodies (ADAs) against etanercept in the hTNF-Tg mice. This result implies that the synergistic effects of etanercept and tacrolimus are not due to secondary effects derived from the suppression of ADA production by tacrolimus but are due to their primary effects. These findings suggest that concomitant treatment with etanercept and tacrolimus may be one of preferable treatment options to control disease activities for patients with rheumatoid arthritis, especially for those with bone resorption.

## 1. Introduction

Rheumatoid arthritis (RA) is one of the most prevalent chronic inflammatory diseases, characterized by progressive articular damage accompanied by extra-articular manifestations such as vasculitis, secondary amyloidosis, and systemic comorbidities [1, 2]. During the past two decades, the management of RA has dramatically changed, owing to the development of efficacious biologics and the establishment of treatment algorithms based on clinical evidence [3, 4]. Currently, conventional synthetic disease-modifying antirheumatic drugs (csDMARDs) are often used in combination with biological DMARDs (bDMARDs) for the treatment of RA with moderate or high disease activity. In many cases, the use of methotrexate is recommended in combination therapy with bDMARDs because of significant clinical evidence and familiarity of rheumatologists with methotrexate as an anchor drug [4, 5].

In Japan, tacrolimus was approved for the treatment of autoimmune diseases, including RA, lupus nephritis, and ulcerative colitis, in 2005, 2007, and 2009, respectively. Tacrolimus has been reported to be effective, and it shows an acceptable safety profile in RA patients with an inadequate response to csDMARDs, including methotrexate [6, 7]. In addition, it has been suggested that add-on tacrolimus with bDMARDs may improve the clinical outcomes, even when patients show inadequate responses to bDMARDs with concomitant methotrexate [8–10]. These findings indicate that the use of tacrolimus may be one of the valuable options for patients with contraindications for or an inadequate response to bDMARDs and/or methotrexate. However, the validity of concomitant therapy of bDMARDs in combination with tacrolimus for the treatment of RA has been less investigated than that in combination with other csDMARDs.

To test the hypothesis that etanercept with concomitant tacrolimus is one of the preferable treatment options to control disease activities for patients with RA, we verified the antirheumatic effects and modes of action of etanercept with concomitant tacrolimus using the hTNF-Tg mouse model, which reflects characteristic features of patients with inadequate responses to anti-TNF-*α* biologics [11].

## 2. Materials and Methods

### 2.1. Drugs

Etanercept (Enbrel®), a recombinant human p75 TNF receptor fused to an IgG constant fragment, was purchased from Pfizer (New York, NY, USA) and diluted with saline (Otsuka Pharmaceutical Factory, Tokushima, Japan) for subcutaneous administration (0.4 mg/mL). Tacrolimus was purchased from MedChemExpress (Monmouth Junction, NJ, USA) and suspended in 1% methylcellulose (Tokyo Chemical Industry, Tokyo, Japan) solution for oral administration (0.3 mg/mL).

### 2.2. Animals

Nine-week-old hemizygous male hTNF-Tg C57BL/6 mice (1006 line) were obtained from Taconic Biosciences (Rensselaer, NY, USA). The mice were housed under a 12 h light-dark cycle at a temperature of 23 ± 3°C and relative humidity of 50 ± 20%, with free access to food and water. All experimental procedures were carried out with approval from the Animal Experimental Ethic Committee of the AYUMI Pharmaceutical Corporation.

### 2.3. Clinical Assessment of Arthritis

Twenty mice were randomized into the following four treatment groups based on their body weights (*n* = 5): a vehicle treatment group and etanercept, tacrolimus, and combined therapy treatment groups. The mice were either subcutaneously injected with 4 mg/kg etanercept twice a week at 3- to 4-day intervals, orally dosed with 3 mg/kg tacrolimus once daily, or administered both drugs for 4 weeks depending on the treatment group. The fore and hind limbs of the mice were inspected twice a week during the 4-week treatment period. Clinical arthritis in each mouse was scored as follows: 0, no swelling; 1, slight swelling; 2, pronounced edematous swelling; and 3, pronounced edematous swelling with joint rigidity. Each limb was graded, resulting in a maximum score of 12 per mouse. On the day after the last dosing, the mice were sacrificed under isoflurane anesthesia and their spleens were excised for flow cytometric analysis following collection of blood from the abdominal vena cava.

### 2.4. Radiographic Assessment of Arthritis

Arthritis-associated radiographic changes in hTNF-Tg mice were evaluated using an UltraFocus digital radiography system (Faxitron Bioptics, Tucson, AZ, USA). Three observers assessed the joint destruction of the limbs in a blinded manner using the following scoring criteria: 0, no joint destruction; 1, minor joint destruction confined to a single digit or to a tarsal bone; 2, marked joint destruction of multiple digits or tarsal bones; and 3, complete destruction of tarsal joints. Four limbs from each mouse were evaluated, resulting in a maximum score of 12 per mouse.

### 2.5. Histopathology

The fore and hind limb specimens from each mouse were fixed in a 10% neutralized formalin buffer solution and decalcified in a 10% ethylenediaminetetraacetate solution until the bones were pliable. Paraffin sections of the limbs were stained with hematoxylin (Merck KGaA, Darmstadt, Germany) and eosin (Merck KGaA) (H&E) following deparaffinization with xylene (Mutokagaku, Tokyo, Japan) and hydration with distilled water. Cartilage damage, bone destruction, synovial hyperplasia, and infiltration of inflammatory cells into the synovial lining in the metacarpal, carpal, metatarsal, tarsal, and phalangeal joints were evaluated using light microscopy. Histopathological scores were assigned based on the following criteria: 0, no change; 1, very slight (slight focal changes); 2, slight (moderate focal changes); 3, moderate (severe focal changes or moderate diffuse changes); and 4, severe (severe diffuse changes), resulting in a maximum score of 16 per mouse for each item.

### 2.6. Determination of Antidrug Antibody; Matrix Metalloproteinase-1, Metalloproteinase-3, and Metalloproteinase-9; and Cytokine Levels

Relative amounts of antidrug antibodies (ADAs) in serum samples were measured by an enzyme-linked immunosorbent assay (ELISA). Briefly, 96-well flat-bottom plates were coated with 1 *μ*g of etanercept, dissolved in phosphate-buffered saline (Nacalai Tesque, Kyoto, Japan), per well. The plates were incubated overnight at 4°C. Washing steps were performed with 50 mM Tris-buffered saline (pH 8.0) and 0.05% Tween 20 (Bethyl Laboratories, Montgomery, TX, USA). Blocking was performed at room temperature for 1 hour with 50 mM Tris-buffered saline (pH 8.0) and 1% BSA (Bethyl Laboratories). Serum dilutions were added to the wells and incubated at room temperature for 1 hour. Specific binding was detected using peroxidase-labeled goat anti-mouse IgG, Human ads (SouthernBiotech, Birmingham, AL, USA) for 30 min at room temperature. The reaction was developed by adding 100 *μ*L of tetramethylbenzidine (Bethyl Laboratories) for 30 min and stopped with 100 *μ*L of 0.18 M H_2_SO_4_ (Bethyl Laboratories). Absorbance was measured at 450 nm using a microplate reader (PHERAstar FSX; BMG Labtech, Ortenberg, Germany). Levels of matrix metalloproteinases (MMPs) in serum samples were determined using a commercially available kit (R&D Systems, Minneapolis, MN, USA) in accordance with the manufacturer's instruction. The serum concentrations of interleukin- (IL-) 23, IL-1*α*, interferon- (IFN-) *γ*, C-C motif chemokine ligand (CCL) 2, IL-12p70, IL-1*β*, IL-10, IL-6, IL-27, IL-17A, IFN-*β*, and granulocyte-macrophage colony-stimulating factor (GM-CSF) were measured using a bead-based multianalyte assay kit (LEGENDplex™ Mouse Inflammation Panel; BioLegend, San Diego, CA, USA) in accordance with the manufacturer's instruction. The minimum detectable concentrations were as follows: IL-23, 12.2 pg/mL; IL-1*α*, 2.44 pg/mL; IFN-*γ*, 2.44 pg/mL; CCL2, 2.44 pg/mL; IL-12p70, 2.44 pg/mL; IL-1*β*, 2.44 pg/mL; IL-10, 2.44 pg/mL; IL-6, 2.44 pg/mL; IL-27, 12.2 pg/mL; IL-17A, 2.44 pg/mL; IFN-*β*, 12.2 pg/mL; and GM-CSF, 2.44 pg/mL.

### 2.7. Flow Cytometry

Following red blood cell lysis, single-cell suspensions of splenocytes from each mouse were incubated with anti-mouse cluster of differentiation 16/32 (CD16/32) monoclonal antibodies (mAbs) (93; BioLegend) to block nonspecific binding of antibodies to Fc receptors. Cells were then stained with anti-mouse CD11b, CD4, programmed cell death protein 1 (PD-1), C-X-C chemokine receptor type 5 (CXCR5), and B220 (CD45R) mAbs (M1/70, GK1.5, 29F.1A12, L138D7, and RA3-6B2, respectively; BioLegend), followed by dead cell staining with ZombieNIR (BioLegend). Data were acquired using a cell sorter SH800S (Sony, Tokyo, Japan) and analyzed with FlowJo software version 10 (Tree Star, Ashland, OR, USA).

### 2.8. Cell Culture

Murine monocyte/macrophage RAW 264.7 cells (Sumitomo Dainippon Pharma, Osaka, Japan) were grown in minimal essential medium alpha (Nacalai Tesque) supplemented with 10% (*v*/*v*) heat-inactivated fetal bovine serum (Biowest, Nuaille, France), 100 U/mL penicillin, and 100 *μ*g/mL streptomycin (Wako Pure Chemical Industries, Osaka, Japan) at 37°C in a humidified atmosphere of 5% CO_2_ in air. For differentiation of osteoclasts, RAW 264.7 cells (5,000 cells per well in a 96-well plate) were cultured in the presence of 10 ng/mL receptor activator of nuclear factor kappa B ligand (RANKL; R&D Systems) for 2 days, and then the cells were stimulated with 10 ng/mL RANKL and 10 ng/mL recombinant human TNF-*α* (R&D Systems) in the presence or absence of the drugs for 3 days.

### 2.9. Cell Viability Assay

Cell viability was determined using a Cell Counting Kit-8 (Dojindo, Kumamoto, Japan) in accordance with the manufacturer's instruction. Briefly, following stimulation with 10 ng/mL RANKL and 10 ng/mL recombinant human TNF-*α* in the presence or absence of the drugs for 3 days, 10 *μ*L of the Cell Counting Kit-8 assay reagent was added to each well, and the absorbance at 450 nm was measured using a microplate reader.

### 2.10. In Vitro Osteoclastogenesis

Osteoclastogenesis was evaluated by quantifying cells positively stained for TRAP. Briefly, the cells on 96-well plates were fixed with a 10% neutral formalin buffer solution for 5 min and then stained using a TRAP staining kit (Cosmo Bio, Tokyo, Japan). Under a light microscope, osteoclasts were observed as TRAP-positive with three or more multinuclear cells, and their total number was counted in each well.

### 2.11. Statistical Analyses

Statistical analyses for multiple comparisons were performed using one-way ANOVA and Dunnet's multiple comparison test or Steel's test depending on the results of Bartlett's test and Kruskal-Wallis test followed by joint-ranking Dunnett's multiple comparison test. Statistical analyses for single comparisons were carried out by an *F*-test followed by Student's *t*-test or Aspin-Welch test (EXSUS; CAC Croit, Tokyo, Japan). Differences were considered statistically significant at a *p* value of less than 0.05.

## 3. Results

### 3.1. Combined Treatment with Etanercept and Tacrolimus Attenuates Arthritis Progression in hTNF-Tg Mice

**Figure 1:**
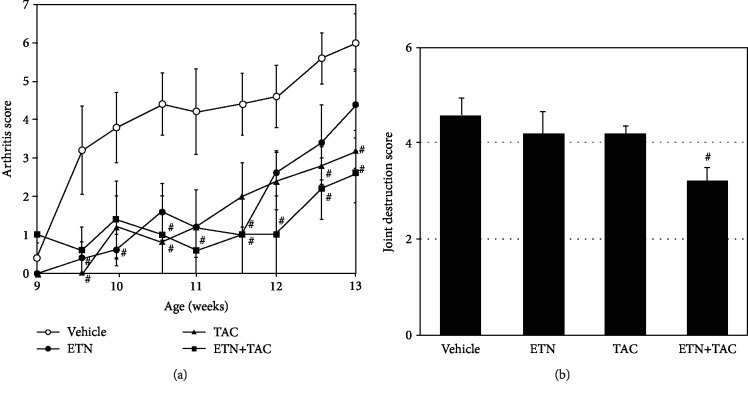
Tacrolimus and its combined treatment with etanercept attenuate the progression of arthritis in hTNF-Tg mice. Etanercept (ETN; 4 mg/kg) was administered subcutaneously twice a week for 4 weeks, and tacrolimus (TAC; 3 mg/kg) was administered orally once daily for 4 weeks. (a) Results are shown as the arthritis score and the mean ± standard error of the mean (SEM) of five animals per group. (b) Arthritis-associated radiographic changes were evaluated in a blinded manner at the end of the treatment period. The joint destruction score and the mean ± SEM of five animals per group are shown. Data are representative of two independent experiments. ^#^*p* < 0.05 compared with the vehicle-treated mice (Dunnet's multiple comparison test).

We investigated the effects of etanercept, tacrolimus, and their combined therapy on the progression of arthritis in hTNF-Tg mice. The first signs of the onset of arthritis in the mice were observed at the age of 9 weeks, and the mean clinical score increased with age in the vehicle treatment group (Figure 1(a)). Treatment with etanercept alone resulted in significant reduction of the mean clinical scores of arthritis at the age of 10 weeks, although no significant difference could be detected between the etanercept and vehicle treatment groups of mice at the age of 12 weeks or later. Tacrolimus and its combination treatment with etanercept apparently attenuated the clinical scores throughout most of the treatment period, with statistical significance (*p* < 0.05). We also evaluated the radiographic changes associated with arthritis in the mice at the end of the treatment period. As shown in Figure 1(b), joint destruction of the limbs was observed in the vehicle treatment group, whereas the combined treatment caused significant suppression of the mean joint destruction score compared with that in the vehicle treatment group (also see Supplemental [Supplementary-material supplementary-material-1]). The mean joint destruction score in each single-drug treatment group was slightly lower than that in the vehicle treatment group, but the differences were not statistically significant. To estimate the side effects caused by the combined treatment, we also evaluated body weight gains and organ weights of the mice in this study. However, we could not find any statistical difference between the groups (Supplemental [Supplementary-material supplementary-material-1]).

**Figure 2:**
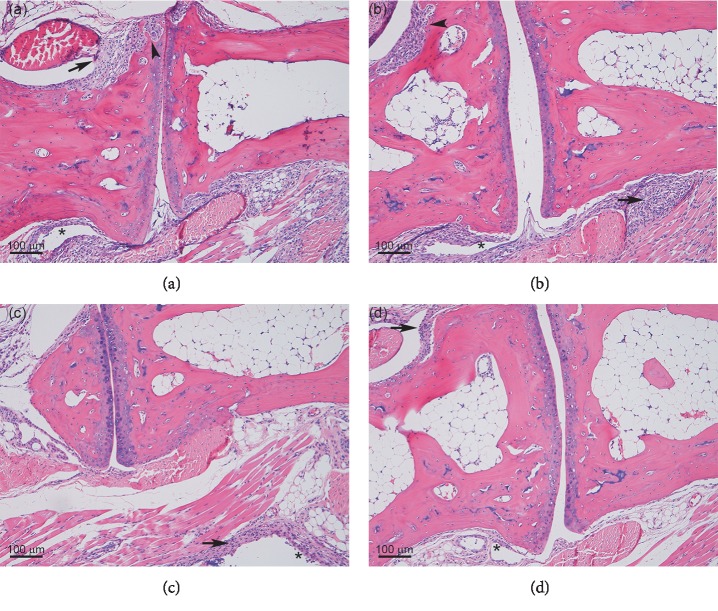
Concomitant treatment with etanercept and tacrolimus attenuates the joint damage in hTNF-Tg mice. Representative H&E-stained sections of the carpal joints from hTNF-Tg mice following treatment with (a) vehicle, (b) etanercept, (c) tacrolimus, and (d) etanercept plus tacrolimus are shown. Arrows, arrowheads, and asterisks in H&E-stained sections indicate infiltration of inflammatory cells, bone destruction, and synovial hyperplasia, respectively.

The histopathological studies showed that hTNF-Tg mice developed polyarthritis, characterized by synovial hyperplasia, inflammation, bone and cartilage destruction, and inflammatory cell infiltration into the synovium (Figure 2), as described previously [11]. As shown in Table 1, single-drug treatment with either etanercept or tacrolimus did not affect the histopathological changes associated with the development of arthritis, whereas their combined therapy apparently tended to inhibit the changes. These findings suggest that the combined therapy enhanced the attenuation of joint damage associated with the progression of arthritis in hTNF-Tg mice.

### 3.2. Tacrolimus Does Not Suppress ADA Development and an Increase in Etanercept-Induced B220^−^CD4^+^CXCR5^+^PD-1^+^ T Follicular Helper Cells

**Figure 3:**
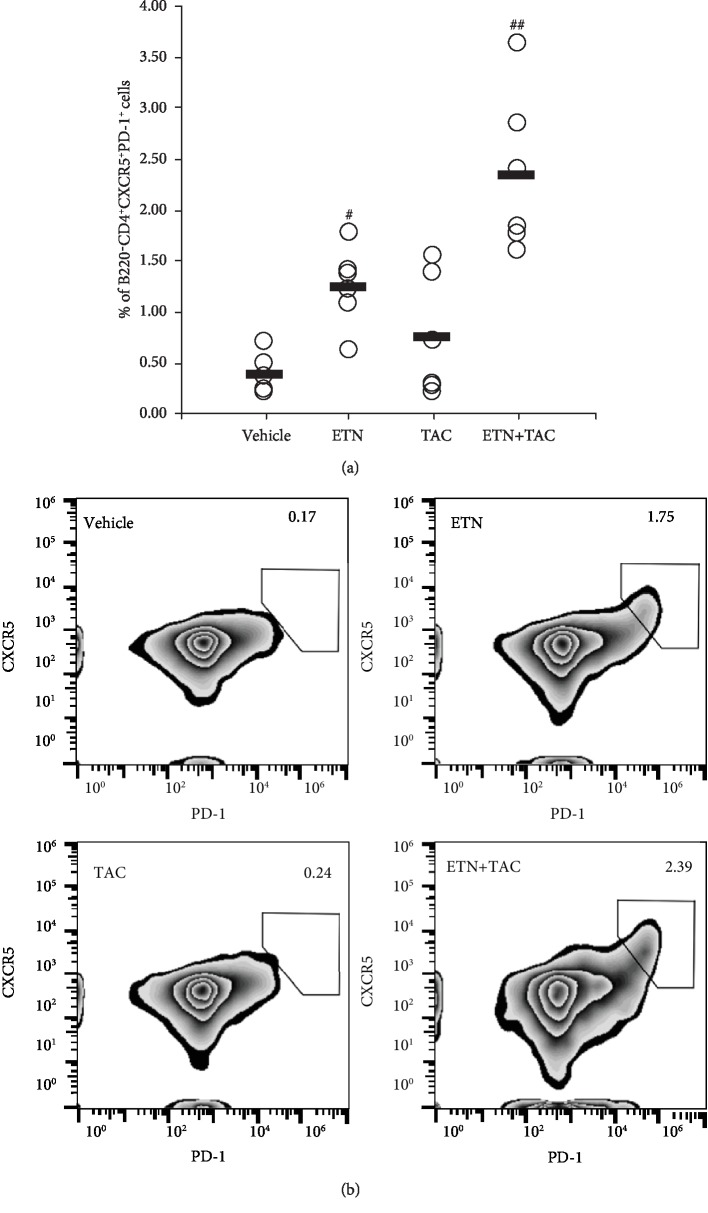
Etanercept increases the number of peripheral B220^−^CD4^+^CXCR5^+^PD-1^+^ Tfh cells in hTNF-Tg mice. Etanercept (ETN; 4 mg/kg) was administered subcutaneously twice a week for 4 weeks, and tacrolimus (TAC; 3 mg/kg) was administered orally once daily for 4 weeks. (a) Percentages of ZombieNIR-negative B220^−^CD4^+^CXCR5^+^PD-1^+^ cells in splenocytes derived from individual mice are shown. Each horizontal bar shows the mean of six animals per group. ^#^*p* < 0.05 and ^##^*p* < 0.01 (Steel's test) compared with the vehicle group. (b) Representative plots of each drug treatment group are shown. Data are representative of two independent experiments.

We examined the presence of ADAs against etanercept in hTNF-Tg mice as the ADAs lead to insufficiency of etanercept. In addition to the measurement of the ADAs, we investigated the number of B220^−^CD4^+^CXCR5^+^PD-1^+^ T follicular helper (Tfh) cells, which play pivotal roles in B cell maturation and humoral immune responses [12], to further examine the effects of the drugs on ADA development. As shown in Table 2, subcutaneous administration of etanercept (4 mg/kg, twice a week for 4 weeks) apparently induced the development of ADA against etanercept, whereas oral treatment with tacrolimus (3 mg/kg, once daily for 4 weeks) did not suppress it. Similarly, the percentage of B220^−^CD4^+^CXCR5^+^PD-1^+^ Tfh cells was significantly higher in the etanercept group than in the vehicle group, whereas tacrolimus did not suppress the increase in etanercept-induced B220^−^CD4^+^CXCR5^+^PD-1^+^ Tfh cells (Figure 3).

### 3.3. Combined Treatment with Etanercept and Tacrolimus Decreases Serum MMP-3 Levels in hTNF-Tg Mice

The serum MMP-3 levels in the hTNF-Tg mice were significantly lower in the combined treatment group than in the vehicle and each single-drug treatment group (Table 3). Although the serum MMP-1 levels were also significantly lower in the etanercept plus tacrolimus group than in the vehicle group, no statistically significant difference was observed in the serum MMP-1 level between each single-drug treatment group and the combined treatment group. Regarding the serum MMP-9 levels, there was no statistically significant difference between the groups. We also examined various inflammatory cytokines, IL-23, IL-1*α*, IFN-*γ*, CCL2, IL-12p70, IL-1*β*, IL-10, IL-6, IL-27, IL-17A, IFN-*β*, and GM-CSF, in serum samples. Although we could detect all these cytokines within the limits of detection, no significant differences were observed between any drug treatment group and the vehicle treatment group (data not shown).

### 3.4. Combined Treatment with Tacrolimus and Etanercept Decreases Peripheral CD11b^high^ Osteoclast Precursors in hTNF-Tg Mice

**Figure 4:**
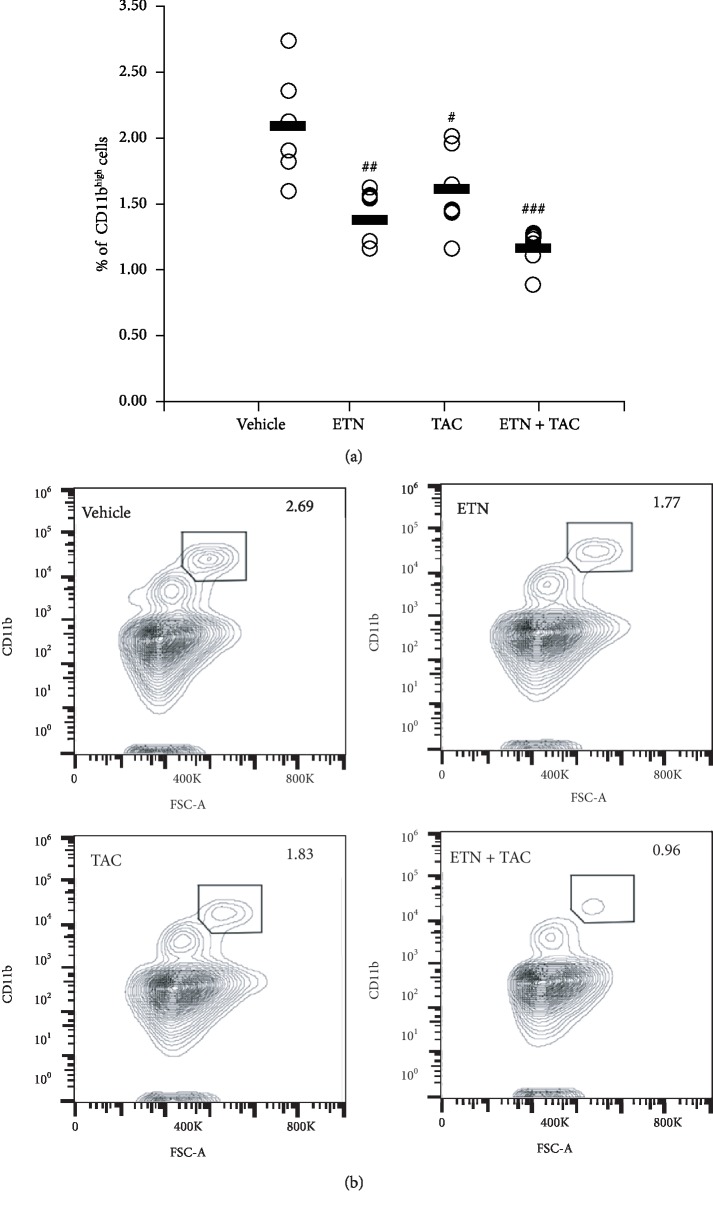
Etanercept and tacrolimus decrease the population of CD11b^high^ peripheral osteoclast precursors in hTNF-Tg mice. Etanercept (ETN; 4 mg/kg) was administered subcutaneously twice a week for 4 weeks, and tacrolimus (TAC; 3 mg/kg) was administered orally once daily for 4 weeks. (a) Percentages of ZombieNIR-negative CD11b^high^ cells in splenic cells derived from individual mice are shown. Each horizontal bar shows the mean of six animals per group. ^#^*p* < 0.05, ^##^*p* < 0.01, and ^###^*p* < 0.001 compared with the vehicle group (Dunnet's multiple comparison test). ^$^*p* < 0.05 compared with the tacrolimus-treated group (Student's *t*-test). (b) Representative plots of each drug treatment group are shown. Data are representative of two independent experiments.

A previous study has demonstrated elevated numbers of peripheral CD11b^high^ osteoclast precursors (OCPs) in hTNF-Tg mice [13]. To evaluate whether each drug treatment could affect the population of peripheral CD11b^high^ OCPs, their numbers were determined by flow cytometric analysis. Treatments with etanercept alone and with tacrolimus alone decreased the percentage of peripheral CD11b^high^ OCPs with significant differences (Figure 4). In addition, the population of peripheral CD11b^high^ OCPs in the combined treatment group was much smaller than those in the other groups.

### 3.5. Etanercept and Tacrolimus Synergistically Affect In Vitro Osteoclastic Differentiation

**Figure 5:**
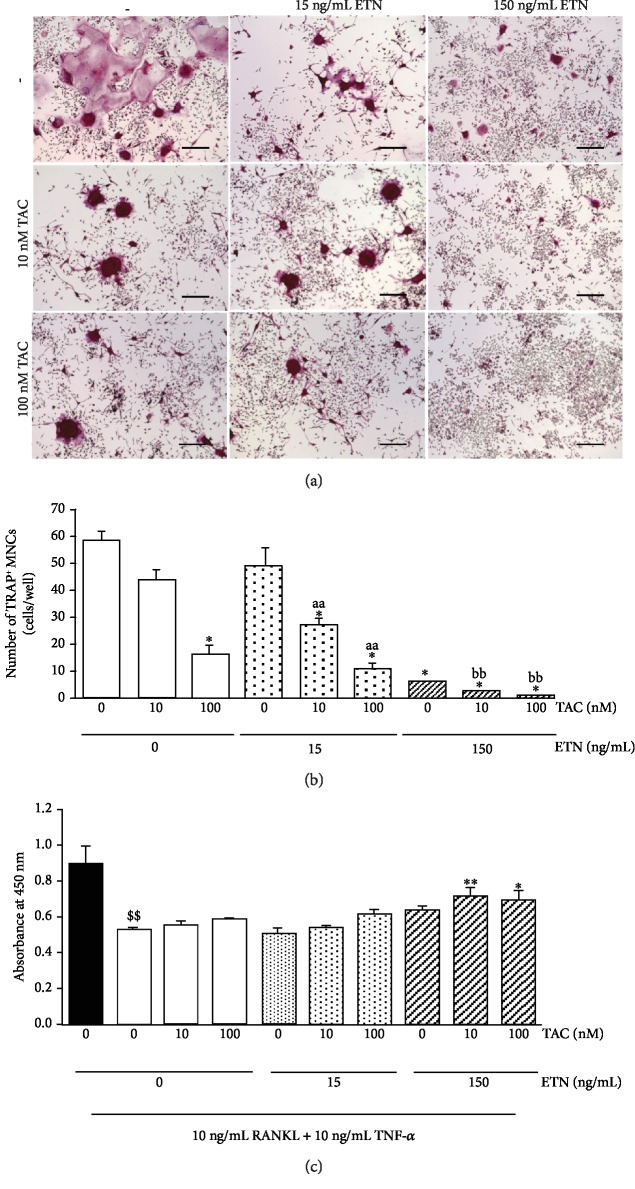
Etanercept (ETN) and tacrolimus (TAC) inhibit the differentiation of RAW 264.7 cells into TRAP-positive multinuclear cells. RAW 264.7 cells were cultured in the presence of 10 ng/mL RANKL for 2 days and then stimulated with 10 ng/mL RANKL and 10 ng/mL recombinant human TNF-*α* in the presence or absence of the drugs for 3 days and stained for TRAP. (a) Representative images of each drug treatment group are shown. Scale bar = 200 *μ*m. (b) Results are shown as the number of TRAP-positive multinuclear cells and the mean ± SEM (*n* = 4).^aa^*p* < 0.01 compared with 15 ng/mL ETN single-drug treatment;^bb^*p* < 0.01 compared with 150 ng/mL ETN single-drug treatment; ^∗^*p* < 0.01 compared with the no-drug treatment group (Dunnett's multiple comparison test). (c) The results of single and concomitant drug treatments on cell viability under the *in vitro* osteoclastogenesis assay. Results are shown as absorbance at 450 nm and the mean ± SEM (*n* = 4). ^$$^*p* < 0.01 compared with the nontreatment group (Aspin-Welch's *t*-test). ^∗^*p* < 0.05 and ^∗∗^*p* < 0.01 compared with the no-drug treatment group (Dunnett's multiple comparison test).

As described earlier, our results suggested that the concomitant treatment with etanercept and tacrolimus seemed to exert synergistic and/or additive effects on osteoclast cell differentiation in the pathologic inflammatory milieu in hTNF-Tg mice. To confirm this, we investigated the effects of each drug and combined treatment on RANKL- and TNF-*α*-induced differentiation of RAW 264.7 cells into TRAP-positive osteoclast-like cells. As shown in Figure 5(a), RAW 264.7 cells stimulated with RANKL and TNF-*α* were fused with one another and differentiated into TRAP-positive multinuclear cells. Treatments with etanercept alone and tacrolimus alone decreased the number of TRAP-positive multinuclear cells in a dose-dependent manner, with significant differences at concentrations of 150 ng/mL and 100 nM, respectively (Figure 5(b)). In addition, the number of TRAP-positive multinuclear cells in the combined treatment group was much lower than that in the single-drug treatment groups at each concentration. These findings were consistent with the preliminary evaluation of TRAP-positive multinuclear cell counts in the joint of hTNF-Tg mice treated with the drugs (Supplemental [Supplementary-material supplementary-material-1]). On the other hand, the RAW 264.7 cell differentiation was not inhibited by etanercept when RAW 264.7 cells were stimulated with RANKL alone (Supplemental [Supplementary-material supplementary-material-1]). The cell survival rate under the same test conditions was also examined, and no reduction in the rate was observed in three treatment groups, compared with the no-drug treatment group, under the drug concentration range used in the test (Figure 5(c)).

## 4. Discussion

This study showed that the combined treatment with etanercept and tacrolimus reduced the number of peripheral CD11b^high^ OCPs and serum MMP-3 levels and attenuated the progression of arthritis in hTNF-Tg mice. In addition, the combined treatment synergistically affected the differentiation of RAW 264.7 cells into TRAP-positive osteoclast-like cells. Furthermore, administration of etanercept to hTNF-Tg mice caused the development of ADAs, whereas tacrolimus did not suppress ADA production. These findings demonstrated that concomitantly administered etanercept and tacrolimus exerted additive and/or synergistic inhibitory effects on the progression of arthritis, largely via suppression of MMP-3 production, CD11b^high^ OCP mobilization, and osteoclast differentiation but did not affect ADA production.

In the present study, we used hTNF-Tg mice to analyze the antirheumatic effects of the drugs because the model shares several clinical, histopathological, immunological, and pathogenetic features of RA in humans [14, 15], and they are reflective of patients with inadequate responses to anti-TNF-*α* biologics [11].

Consistent with the findings of a previous study [11], etanercept monotherapy inhibited the progression of clinical arthritis in the mice; however, the efficacy of the drug was attenuated with the elapsed days. Recently, Arnoult et al. have reported that a single intravenous injection of anti-TNF-*α* mAbs induced the development of ADAs in C57BL/6 wild-type mice [16]. Their findings also revealed that ADA production was induced by a TNF-*α*/anti-TNF-*α* mAb immune complex. This report led us to examine whether ADAs were induced against etanercept because etanercept forms an immune complex with TNF-*α* in hTNF-Tg mice, and we found the ADAs in the serum from the etanercept treatment group. Based on this finding, it seems reasonable to assume that one of the reasons for the insufficiency of etanercept observed in this study was the production of ADAs against etanercept. In addition, we could not detect inhibitory effects of tacrolimus on ADA production, and it also appeared that tacrolimus did not affect the number of peripheral B220^−^CD4^+^CXCR5^+^PD-1^+^ Tfh cells, which are induced by etanercept. These findings suggest that tacrolimus does not inhibit ADA production in the inflammatory milieu in hTNF-Tg mice.

ADAs often attenuate the clinical efficacies of therapeutic biologics by inhibiting or neutralizing their activities and reducing the drugs' half-lives. From this point of view, drugs that suppress ADA development, such as methotrexate, are preferable concomitant drugs in RA treatment [17]. However, ADAs against etanercept have been suggested to not be neutralizing antibodies and to not be linked to clinical responses [18–20]. Therefore, we believe that even though it could not suppress the development of ADAs, tacrolimus may be one of the good treatment options as an alternative to methotrexate for concomitant therapy with etanercept for RA patients with intolerance to methotrexate.

Previous studies have suggested that several MMPs are involved in facilitation of synovial inflammation, cartilage destruction, and bone resorption in hTNF-Tg mice [20]. Although Zwerina et al. have demonstrated that the pharmacological blockade of TNF-*α* by infliximab, a chimeric human/mouse anti-TNF-*α* mAb, decreases the serum levels of IL-1 in hTNF-Tg mice [11], we could not detect the reduction of IL-1 levels in the etanercept treatment group. This may be partly due to differences in the mechanisms of action between infliximab and etanercept; infliximab but not etanercept induces the cell cycle arrest and regulates the cytokine expression by outside-to-inside signals through transmembrane TNF-*α* [21]. Consistent with our findings, Schett et al. have clearly demonstrated that forced expression of the tissue inhibitor of MMP-1 [20], which is an endogenous inhibitor of MMP-3 [22], improved the clinical signs and attenuated the joint destruction without affecting the serum levels of inflammatory cytokines in mice.

Bond et al. have suggested that the activation of nuclear factor kappa B (NF-*κ*B), together with activator protein 1 (AP-1), is essential for the growth factor- or inflammatory cytokine-induced MMP-3 synthesis [23]. NF-*κ*B activation is a common feature of RA synovium, and it is often observed in peripheral blood mononuclear cells, peripheral blood cells, and monocytes from patients with RA [24, 25]. Previous reports have demonstrated that NF-*κ*B activation was markedly induced by TNF-*α* in those cells and blocked by anti-TNF-*α* biologics [26]. In addition, Migita et al. have demonstrated that tacrolimus could attenuate AP-1 transcriptional activation by abrogating c-Jun N-terminal kinase 1/2 phosphorylation in fibroblast-like synoviocytes from patients with RA [27]. Taken together, etanercept administered concomitantly with tacrolimus exerts enhanced inhibitory effects on MMP-3 production by blocking both the transcription factors, NF-*κ*B and AP-1, essential for MMP-3 synthesis.

To further examine the effects of the etanercept plus tacrolimus concomitant treatment on osteoclastogenesis, we determined the frequency of CD11b^high^ OCPs in the population of splenocytes, based on an earlier finding by Li et al. [13]. The results revealed that peripheral CD11b^high^ OCPs were apparently diminished by the combined treatment, whereas each single-drug treatment only showed modest anti-OCP effects. A previous report has shown that TNF-*α* affects the recruitment of CD11b^high^ OCPs from the bone marrow into the periphery without affecting their proliferation, differentiation, and apoptosis [13]. Thus, the etanercept-induced decrease in peripheral CD11b^high^ OCPs could be due to their reduced mobilization from the bone marrow to the circulation, caused by neutralization of TNF-*α*. Consistent with our findings, Uster et al. recently reported that etanercept suppressed the joint damage in an antigen-induced murine arthritis model not via direct inhibition of synovial inflammation but via reduction of the OCP supply from the bone marrow to the periphery [28]. In this study, we could not determine why tacrolimus reduced the peripheral CD11b^high^ OCP population in synergy with etanercept. Although we hypothesized that tacrolimus might affect the production of stromal cell-derived factor 1 and chemokine (C-X3-C motif) ligand 1, which regulates the migration of OCPs [29], we could not detect these chemoattractants, not at least in the mouse serum (data not shown).

It is known that osteoclast differentiation is efficiently induced by sustained activation of the nuclear factor of activated T cell (NFAT) c1 through its synergy with two signaling pathways [30], the TNF receptor signaling pathway and the RANKL-RANK pathway. In fact, Takayanagi et al. have demonstrated that calcineurin inhibitors such as tacrolimus and cyclosporine A inhibit the RANKL-induced nuclear translocation of NFATc1 in bone marrow-derived monocyte/macrophage precursor cells (BMMs) and their differentiation into TRAP-positive multinuclear cells [30]. Besides activating the intracellular TNF-*α* receptor signaling pathway in BMMs, TNF-*α* also indirectly facilitates the differentiation of BMMs into osteoclasts by enhancing the expression of RANKL and macrophage colony-stimulating factor in several types of cells [31, 32]. Therefore, the synergistic inhibitory effects of etanercept and tacrolimus on osteoclastogenesis, observed in the present study, may be due to blocking of both the TNF receptor and RANKL-RANK pathways, resulting in complete inhibition of NFATc1 activation.

## 5. Conclusions

In conclusion, concomitant treatment with etanercept and tacrolimus synergistically attenuated the progression of arthritis in hTNF-Tg mice. Although further studies are needed to verify the molecular mechanisms of action of the combined treatment, the inhibition of the MMP-3 production, CD11b^high^ OCP mobilization, and osteoclast differentiation may represent the characteristic features of the concomitant therapy of etanercept with tacrolimus. These findings suggest that concomitant treatment with etanercept and tacrolimus may be one of preferable treatment options to control disease activities for patients with RA, especially for those with inadequate responses to anti-TNF-*α* biologics or with bone resorption.

## Figures and Tables

**Table 1:** Etanercept with concomitantly administered tacrolimus attenuates the inflammatory cell infiltration into the synovial lining in hTNF-Tg mice.

Parameter	Vehicle	ETN	TAC	ETN+TAC
Bone destruction	4.2 ± 0.4	4.2 ± 0.6	5.2 ± 0.5	3.0 ± 0.7
Cartilage damage	4.6 ± 0.4	4.8 ± 0.6	5.0 ± 0.7	3.8 ± 0.9
Synovial hyperplasia	7.2 ± 0.7	8.8 ± 0.2	8.2 ± 0.8	8.0 ± 0.8
Infiltration of inflammatory cells	10.6 ± 0.6	10.4 ± 0.5	9.2 ± 0.4	8.6 ± 0.4^∗^

H&E-stained sections of the limb joints from hTNF-Tg mice were scored as follows: 0: no change; 1: very slight; 2: slight; 3: moderate; 4: severe, with a maximum score of 16 per mouse for each item. Data are representative of two independent experiments and presented as mean ± SEM of five animals per group. ^∗^*p* < 0.05 compared with the vehicle-treated group (*joint-ranking Dunnett's* multiple comparison test). ETN: etanercept; TAC: tacrolimus.

**Table 2:** Etanercept induces ADA production in hTNF-Tg mice.

	Vehicle	ETN	TAC	ETN+TAC
Relative serum ADA level (O. D. at 450 nm)	−0.01 ± 0.00	1.25 ± 0.06^∗^	−0.01 ± 0.00	1.66 ± 0.50^∗^

Etanercept (ETN; 4 mg/kg) was administered subcutaneously twice a week for 4 weeks, and tacrolimus (TAC; 3 mg/kg) was administered orally once daily for 4 weeks. Relative amounts of serum ADAs against etanercept were measured at the end of the dosing period by ELISA. Data are representative of two independent experiments and are presented as mean ± SEM of five animals per group. ^∗^*p* < 0.05 compared with the vehicle group (Steel's test).

**Table 3:** Combined treatment with etanercept and tacrolimus synergistically reduces the serum MMP-3 levels in hTNF-Tg mice.

	Vehicle	ETN	TAC	ETN+TAC
Serum MMP-3 level (ng/mL)	3.96 ± 0.94	5.18 ± 1.11	2.31 ± 0.23	1.62 ± 0.18^#,$,∗^
Serum MMP-1 level (ng/mL)	508.38 ± 35.56	364.75 ± 14.97	363.98 ± 54.02	338.45 ± 33.99^#^
Serum MMP-9 level (ng/mL)	1.29 ± 0.14	1.40 ± 0.12	1.22 ± 0.12	1.12 ± 0.10

Etanercept (ETN; 4 mg/kg) was administered subcutaneously twice a week for 4 weeks. Tacrolimus (TAC; 3 mg/kg) was administered orally once daily for 4 weeks. The serum MMP-1, MMP-3, and MMP-9 levels were measured by ELISA. Data are representative of two independent experiments and presented as mean ± SEM of five animals per group. ^#^*p* < 0.05 compared with the vehicle group (Dunnett's multiple comparison test). ^$^*p* < 0.05 and ^∗^*p* < 0.05 compared with the tacrolimus-treated group (Student's *t*-test) and the etanercept-treated group (Aspin-Welch's *t*-test), respectively.

## Data Availability

The data used to support the findings of this study are available from the corresponding author upon request.

## Supplementary Materials

Supplementary Materials Supplemental Figure 1: representative X-ray images of the hind limbs of hTNF-Tg mice. Supplemental Figure 2: body weight increments and organ weights in hTNF-Tg mice. Supplemental Figure 3: the effects on RANKL-induced RAW 264.7 cell differentiation. Supplemental Table 1: the number of TRAP-positive MNCs at the articular surface of the joints.

## References

[1] McInnes I. B., Schett G. (2017). Pathogenetic insights from the treatment of rheumatoid arthritis. *The Lancet*.

[2] Smolen J. S., Aletaha D., McInnes I. B. (2016). Rheumatoid arthritis. *The Lancet*.

[3] Nam J. L., Takase-Minegishi K., Ramiro S. (2017). Efficacy of biological disease-modifying antirheumatic drugs: a systematic literature review informing the 2016 update of the EULAR recommendations for the management of rheumatoid arthritis. *Annals of the Rheumatic Diseases*.

[4] Smolen J. S., Landewé R., Bijlsma J. (2017). EULAR recommendations for the management of rheumatoid arthritis with synthetic and biological disease-modifying antirheumatic drugs: 2016 update. *Annals of the Rheumatic Diseases*.

[5] Singh J. A., Saag K. G., Bridges S. L. (2016). 2015 American College of Rheumatology guideline for the treatment of rheumatoid arthritis. *Arthritis & Rhematology*.

[6] Kitahama M., Nakajima A., Inoue E., Taniguchi A., Momohara S., Yamanaka H. (2014). Efficacy of adjunct tacrolimus treatment in patients with rheumatoid arthritis with inadequate responses to methotrexate. *Modern Rheumatology*.

[7] Motomura H., Matsushita I., Seki E., Mine H., Kimura T. (2014). Inhibitory effect of tacrolimus on progression of joint damage in patients with rheumatoid arthritis. *International Journal of Rheumatic Diseases*.

[8] Kaneshiro S., Ebina K., Hirao M. (2017). The efficacy and safety of additional administration of tacrolimus in patients with rheumatoid arthritis who showed an inadequate response to tocilizumab. *Modern Rheumatology*.

[9] Naniwa T., Watanabe M., Banno S., Maeda T. (2009). Adding low dose tacrolimus in rheumatoid arthritis patients with an inadequate response to tumor necrosis factor inhibitor therapies. *Rheumatology International*.

[10] Takeuchi T., Ishida K., Shiraki K., Yoshiyasu T. (2018). Safety and effectiveness of tacrolimus add-on therapy for rheumatoid arthritis patients without an adequate response to biological disease-modifying anti-rheumatic drugs (DMARDs): post-marketing surveillance in Japan. *Modern Rheumatology*.

[11] Zwerina J., Hayer S., Tohidast-Akrad M. (2004). Single and combined inhibition of tumor necrosis factor, interleukin-1, and RANKL pathways in tumor necrosis factor-induced arthritis: effects on synovial inflammation, bone erosion, and cartilage destruction. *Arthritis and Rheumatism*.

[12] Crotty S. (2011). Follicular helper CD4 T cells (TFH). *Annual Review of Immunology*.

[13] Li P., Schwarz E. M., O'Keefe R. J. (2004). Systemic tumor necrosis factor *α* mediates an increase in peripheral CD11b^high^ osteoclast precursors in tumor necrosis factor *α* -transgenic mice. *Arthritis and Rheumatism*.

[14] Keffer J., Probert L., Cazlaris H. (1991). Transgenic mice expressing human tumour necrosis factor: a predictive genetic model of arthritis. *The EMBO Journal*.

[15] Kontoyiannis D., Pasparakis M., Pizarro T. T., Cominelli F., Kollias G. (1999). Impaired on/off regulation of TNF biosynthesis in mice lacking TNF AU-rich elements: implications for joint and gut-associated immunopathologies. *Immunity*.

[16] Arnoult C., Brachet G., Cadena Castaneda D. (2017). Crucial role for immune complexes but not FcRn in immunization against anti-TNF-*α* antibodies after a single injection in mice. *The Journal of Immunology*.

[17] Krieckaert C. L., Nurmohamed M. T., Wolbink G. J. (2012). Methotrexate reduces immunogenicity in adalimumab treated rheumatoid arthritis patients in a dose dependent manner. *Annals of the Rheumatic Diseases*.

[18] Dore R. K., Mathews S., Schechtman J. (2007). The immunogenicity, safety, and efficacy of etanercept liquid administered once weekly in patients with rheumatoid arthritis. *Clinical and Experimental Rheumatology*.

[19] van Schouwenburg P. A., Rispens T., Wolbink G. J. (2013). Immunogenicity of anti-TNF biologic therapies for rheumatoid arthritis. *Nature Reviews Rheumatology*.

[20] Schett G., Hayer S., Tohidast-Akrad M. (2001). Adenovirus-based overexpression of tissue inhibitor of metalloproteinases 1 reduces tissue damage in the joints of tumor necrosis factor *α* transgenic mice. *Arthritis and Rheumatism*.

[21] Mitoma H., Horiuchi T., Hatta N. (2005). Infliximab induces potent anti-inflammatory responses by outside-to-inside signals through transmembrane TNF-*α*. *Gastroenterology*.

[22] Van Meurs J., Van Lent P., Stoop R. (1999). Cleavage of aggrecan at the Asn^341^-Phe^342^ site coincides with the initiation of collagen damage in murine antigen-induced arthritis: a pivotal role for stromelysin 1 in matrix metalloproteinase activity. *Arthritis and Rheumatism*.

[23] Bond M., Baker A. H., Newby A. C. (1999). Nuclear factor *κ*B activity is essential for matrix metalloproteinase-1 and -3 upregulation in rabbit dermal fibroblasts. *Biochemical and Biophysical Research Communications*.

[24] Asahara H., Asanuma M., Ogawa N., Nishibayashi S., Inoue H. (1995). High DNA-binding activity of transcription factor NF-kappa B in synovial membranes of patients with rheumatoid arthritis. *Biochemistry and Molecular Biology International*.

[25] Collantes E., Valle Blázquez M., Mazorra V., Macho A., Aranda E., Muñoz E. (1998). Nuclear factor-*κ*B activity in T cells from patients with rheumatic diseases: a preliminary report. *Annals of the Rheumatic Diseases*.

[26] Dichamp I., Bourgeois A., Dirand C., Herbein G., Wendling D. (2007). Increased nuclear factor-kappaB activation in peripheral blood monocytes of patients with rheumatoid arthritis is mediated primarily by tumor necrosis factor-alpha. *The Journal of Rheumatology*.

[27] Migita K., Miyashita T., Maeda Y. (2005). FK506 suppresses the stimulation of matrix metalloproteinase 13 synthesis by interleukin-1*β* in rheumatoid synovial fibroblasts. *Immunology Letters*.

[28] Uster S., Coelho F. M., Aeberli D. (2018). TNF*α* blockade mediates bone protection in antigen-induced arthritis by reducing osteoclast precursor supply. *Bone*.

[29] Koizumi K., Saitoh Y., Minami T. (2009). Role of CX3CL1/fractalkine in osteoclast differentiation and bone resorption. *The Journal of Immunology*.

[30] Takayanagi H., Kim S., Koga T. (2002). Induction and activation of the transcription factor NFATc1 (NFAT2) integrate RANKL signaling in terminal differentiation of osteoclasts. *Developmental Cell*.

[31] Nakano K., Okada Y., Saito K. (2007). Rheumatoid synovial endothelial cells produce macrophage colony-stimulating factor leading to osteoclastogenesis in rheumatoid arthritis. *Rheumatology*.

[32] Zhang Y. H., Heulsmann A., Tondravi M. M., Mukherjee A., Abu-Amer Y. (2001). Tumor necrosis factor-alpha (TNF) stimulates RANKL-induced osteoclastogenesis via coupling of TNF type 1 receptor and RANK signaling pathways. *The Journal of Biological Chemistry*.

