# Supplementary material for: Concomitant Treatment with Etanercept and Tacrolimus Synergistically Attenuates Arthritis Progression via Inhibition of Matrix Metalloproteinase-3 Production and Osteoclastogenesis in Human TNF-α Transgenic Mice
Source: Mediators Inflamm. 2019 Dec 17;2019:4176974. doi: 10.1155/2019/4176974 (PMC6942915; doi:10.1155/2019/4176974)
Supplement: Supplementary Materials — Supplemental Figure 1: representative X-ray images of the hind limbs of hTNF-Tg mice. Supplemental Figure 2: body weight increments and organ weights in hTNF-Tg mice. Supplemental Figure 3: the effects on RANKL-induced RAW 264.7 cell differentiation. Supplemental Table 1: the number of TRAP-positive MNCs at the articular surface of the joints. [file 4176974.f1.pptx]

## Slide 1
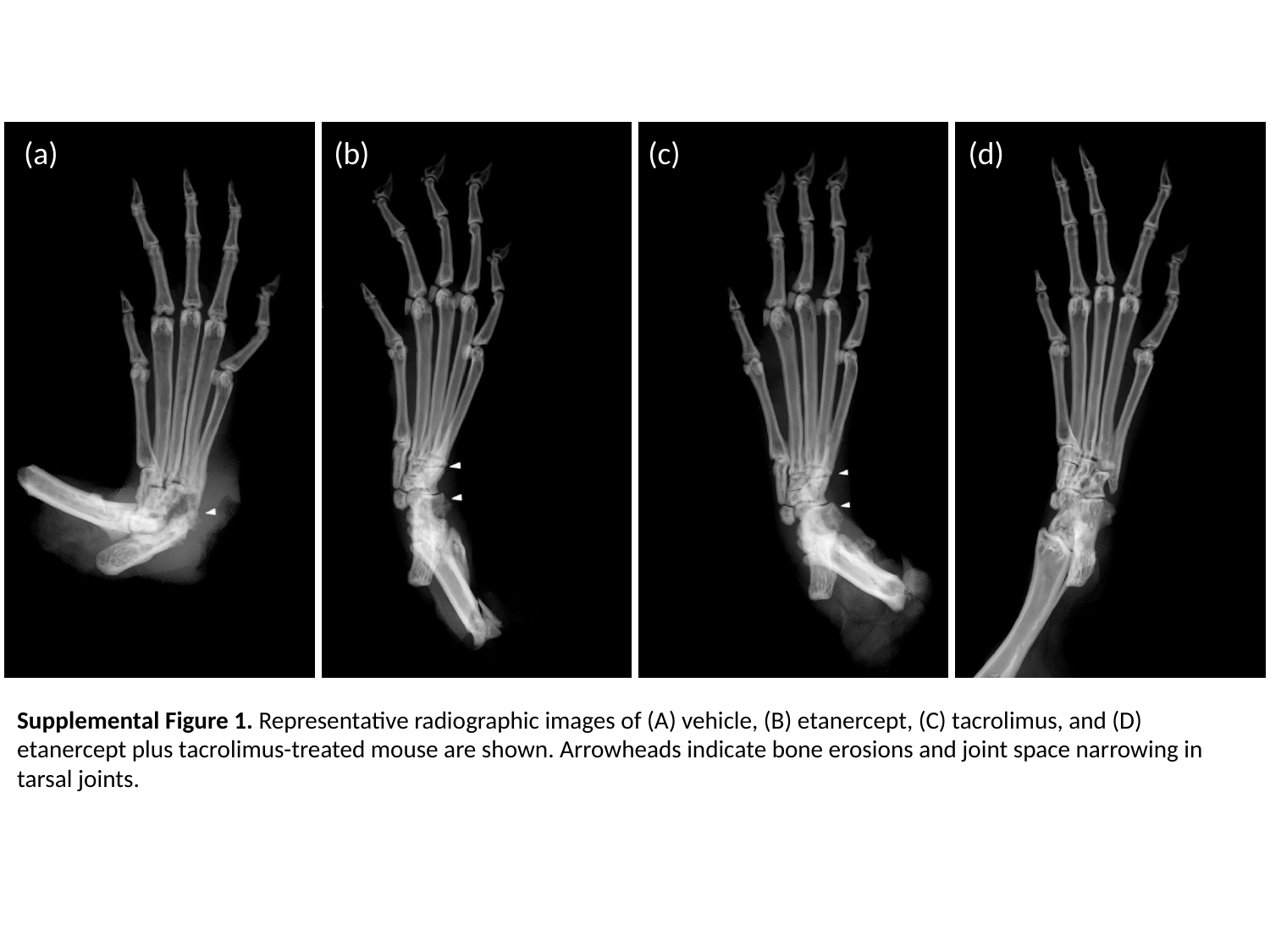

(a)
(b)
(c)
(d)
Supplemental Figure 1. Representative radiographic images of (A) vehicle, (B) etanercept, (C) tacrolimus, and (D) etanercept plus tacrolimus-treated mouse are shown. Arrowheads indicate bone erosions and joint space narrowing in tarsal joints.

## Slide 2
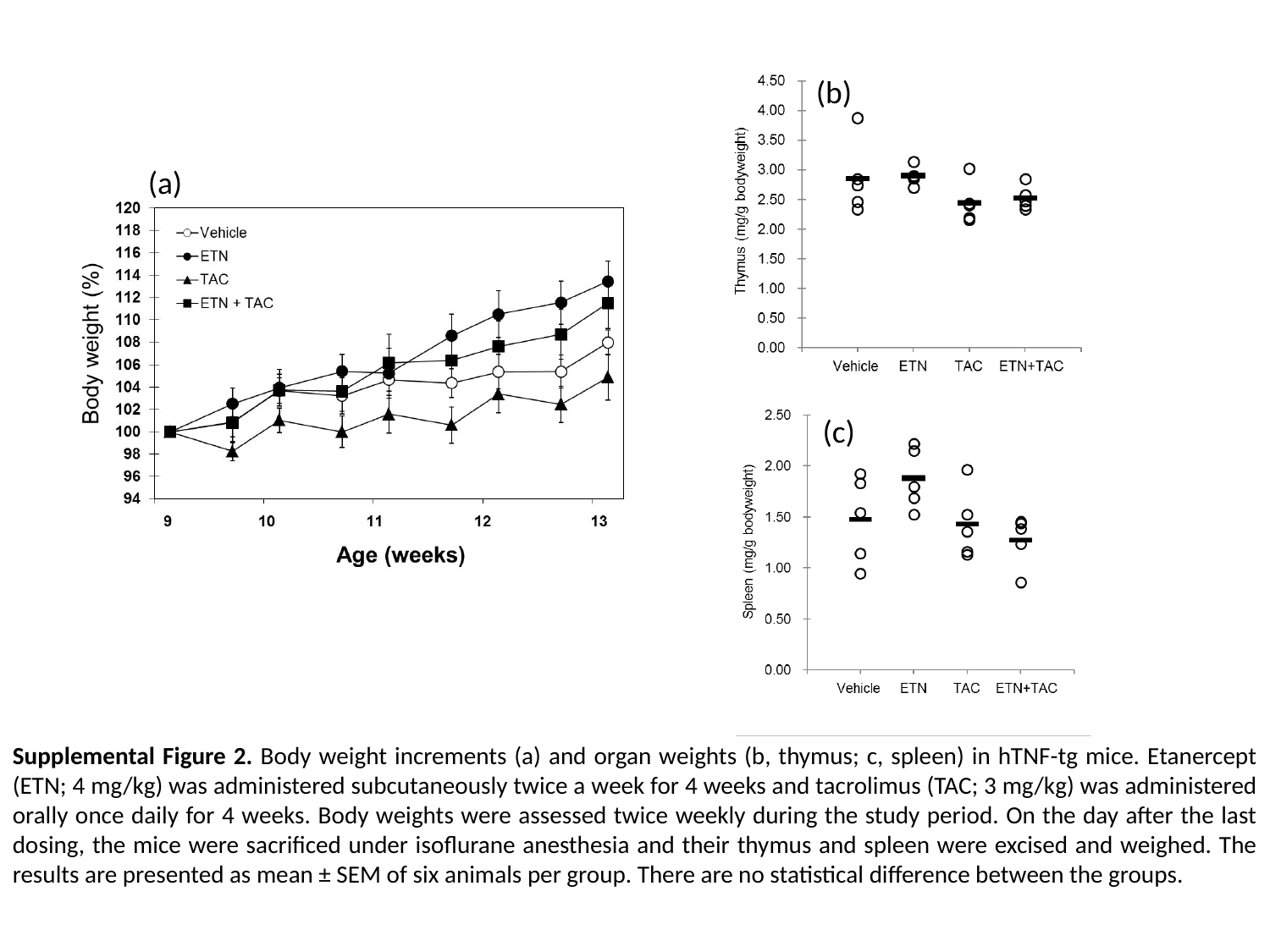

(b)
(a)
(c)
Supplemental Figure 2. Body weight increments (a) and organ weights (b, thymus; c, spleen) in hTNF-tg mice. Etanercept (ETN; 4 mg/kg) was administered subcutaneously twice a week for 4 weeks and tacrolimus (TAC; 3 mg/kg) was administered orally once daily for 4 weeks. Body weights were assessed twice weekly during the study period. On the day after the last dosing, the mice were sacrificed under isoflurane anesthesia and their thymus and spleen were excised and weighed. The results are presented as mean ± SEM of six animals per group. There are no statistical difference between the groups.

## Slide 3
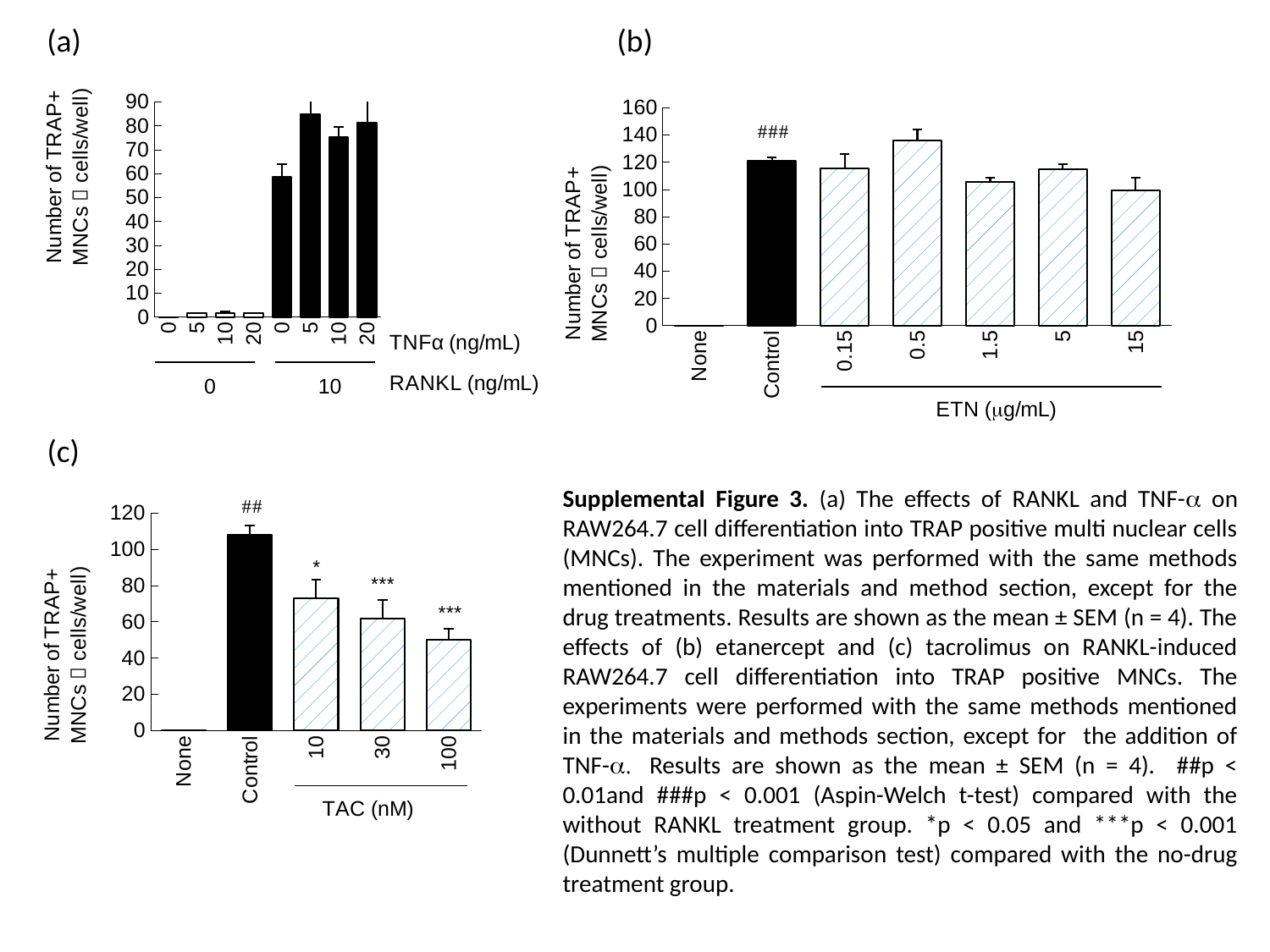

(a)
(b)
### Chart
| Category | |
|---|---|
| None | 0.0 |
| Control | 120.875 |
| 0.15 | 115.5 |
| 0.5 | 135.75 |
| 1.5 | 105.5 |
| 5 | 115.0 |
| 15 | 99.0 |
### Chart
| Category | |
|---|---|
| 0 | 0.0 |
| 5 | 1.5 |
| 10 | 1.75 |
| 20 | 1.5 |
| 0 | 58.75 |
| 5 | 84.75 |
| 10 | 75.25 |
| 20 | 81.25 |C
(c)
### Chart
| Category | |
|---|---|
| None | 0.0 |
| Control | 108.0625 |
| 10 | 73.0 |
| 30 | 61.75 |
| 100 | 50.25 |Supplemental Figure 3. (a) The effects of RANKL and TNF-a on RAW264.7 cell differentiation into TRAP positive multi nuclear cells (MNCs). The experiment was performed with the same methods mentioned in the materials and method section, except for the drug treatments. Results are shown as the mean ± SEM (n = 4). The effects of (b) etanercept and (c) tacrolimus on RANKL-induced RAW264.7 cell differentiation into TRAP positive MNCs. The experiments were performed with the same methods mentioned in the materials and methods section, except for the addition of TNF-a. Results are shown as the mean ± SEM (n = 4). ##p < 0.01and ###p < 0.001 (Aspin-Welch t-test) compared with the without RANKL treatment group. *p < 0.05 and ***p < 0.001 (Dunnett’s multiple comparison test) compared with the no-drug treatment group.

## Slide 4
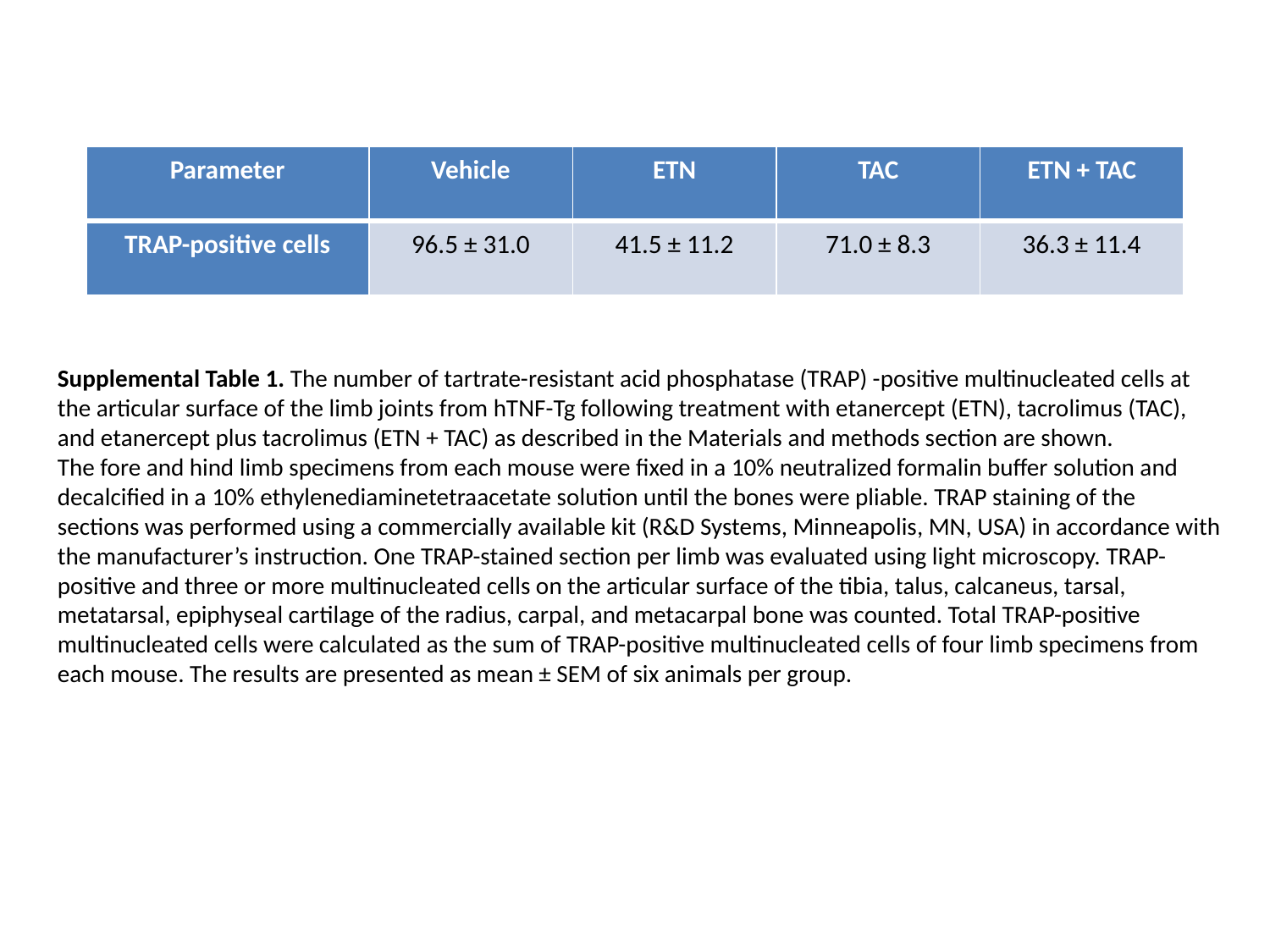

| Parameter | Vehicle | ETN | TAC | ETN + TAC |
| --- | --- | --- | --- | --- |
| TRAP-positive cells | 96.5 ± 31.0 | 41.5 ± 11.2 | 71.0 ± 8.3 | 36.3 ± 11.4 |
Supplemental Table 1. The number of tartrate-resistant acid phosphatase (TRAP) -positive multinucleated cells at the articular surface of the limb joints from hTNF-Tg following treatment with etanercept (ETN), tacrolimus (TAC), and etanercept plus tacrolimus (ETN + TAC) as described in the Materials and methods section are shown.
The fore and hind limb specimens from each mouse were fixed in a 10% neutralized formalin buffer solution and decalcified in a 10% ethylenediaminetetraacetate solution until the bones were pliable. TRAP staining of the sections was performed using a commercially available kit (R&D Systems, Minneapolis, MN, USA) in accordance with the manufacturer’s instruction. One TRAP-stained section per limb was evaluated using light microscopy. TRAP-positive and three or more multinucleated cells on the articular surface of the tibia, talus, calcaneus, tarsal, metatarsal, epiphyseal cartilage of the radius, carpal, and metacarpal bone was counted. Total TRAP-positive multinucleated cells were calculated as the sum of TRAP-positive multinucleated cells of four limb specimens from each mouse. The results are presented as mean ± SEM of six animals per group.
